# Optimizing stress ulcer prophylaxis practices and reducing associated costs in intensive care units: a non-randomized controlled study

**DOI:** 10.3389/jpps.2025.14295

**Published:** 2025-04-10

**Authors:** Yunus Emre Ayhan, Güneş Eskidemir, Ayşe Gül Koçoğlu Kınal, Nilay Aksoy

**Affiliations:** ^1^ Prof. Dr. Cemil Taşcıoğlu City Hospital, Department of Clinical Pharmacy, Istanbul, Türkiye; ^2^ Gaziosmanpaşa Education and Research Hospital, Intensive Care, İstanbul, Türkiye; ^3^ Dr. Siyami Ersek Thoracic and Cardiovascular Surgery Training and Research Hospital, Intensive Care, Istanbul, Türkiye; ^4^ Altınbaş University, School of Pharmacy, Department of Clinical Pharmacy, Istanbul, Türkiye

**Keywords:** stress ulcer prophylaxis, proton pump inhibitor, intensive care unit, clinical pharmacist, cost-saving

## Abstract

**Objective:**

This study evaluated the use of stress ulcer prophylaxis (SUP), assessed the costs associated with inappropriate use, and highlighted the impact of clinical pharmacists on improving adherence to the SUP guidelines.

**Method:**

A prospective, non-randomized controlled study was carried out in two intensive care units (ICUs) of a training and research hospital between 1 June 2023 and 1 December 2023. Routine care services were provided for the observation group (OG) patients. In the guideline group (GG) patients, SUP management and routine care were performed according to ASHP guidelines. The physician and clinical pharmacist jointly evaluated the patients to determine the suitability of their SUP indications. Adherence rates to ASHP guidelines and the costs associated with nonadherence were evaluated.

**Results:**

A total of 196 patients were included in the study: 121 in the OG and 75 in the GG. A total of 54.6% of the patients were male, and the reason for hospitalization was mainly surgery (52.6%). SUP use was higher in OG (100%) than in GG (42.6%) (p < 0.001). The indication rate according to the ASHP guidelines was significantly higher in the GG group (100%) than in the OG group (54.5%) (p < 0.001). Dosage form adherence was significantly lower in the OG (0%) than in the GG (100%) (p < 0.001). The costs associated with proton pump inhibitor use for inappropriate indications and incorrect dosage forms were $60 versus $0 (p < 0.001) and $321 versus $0 (p < 0.001) in OG and GG, respectively. Overall, cost savings of $327 were achieved in the GG group.

**Conclusion:**

Inappropriate SUP use is common in the ICUs. Adequate adherence to guidelines and proactive involvement of clinical pharmacists may reduce inappropriate SUP in ICUs and the associated costs.

## Introduction

Patients admitted to the intensive care unit (ICU) are at increased risk of developing gastrointestinal (GI) bleeding due to stress, which in turn increases the likelihood of morbidity and mortality. Stress-related mucosal injury can develop in the stomach and duodenum and can progress to ulceration during the first 4–5 days after admission [1, 2]. Stress ulcer prophylaxis (SUP) is one of the most used strategies to prevent stress ulcers in ICUs worldwide [3, 4]. Two major independent predictors for clinically significant GI bleeding in ICU patients were identified in a multicenter study. The first was invasive mechanical ventilation (MV) for 48 h or longer (odds ratio [OR] for bleeding, 15.6; 95% confidence interval [CI], 3–80), and the second was coagulopathy (OR, 4.5; 95% CI, 1.8–10.3) [5]. These risk factors are also recognized as significant in the SUP therapeutic guidelines of the American Society of Health-System Pharmacists ASHP [1].

Proton pump inhibitors (PPIs) are among the most used medications in critically ill patients for SUP. However, the inappropriate and inconsistent use of PPIs in ICUs has increased unnecessary costs, increased risks related to adverse drug reactions, and possible complications such as pneumonia, *Clostridioides difficile* infections, hypomagnesemia, and bone fractures [6]. Studies have shown that a significant percentage of patients in ICUs have been receiving PPIs without appropriate indications [6–8]. In contrast, only 59.4% of patients had an appropriate indication according to the ASHP guidelines, which indicates considerable overuse of PPIs in ICUs. Moreover, 38.5% of patients received inappropriate prophylaxis at the time of admission, with 44% receiving SUP for a period longer than appropriate [6].

A few studies have assessed adherence to SUP guidelines and institutional standards under the surveillance of a pharmacist. The results of these studies implied that pharmacist supervision reduced the inappropriate use of SUP in patients and its associated healthcare costs [9–12]. One of these studies noted that the intervention and adjustment of pharmacists reduced the incidence of inappropriate use of SUP and its associated costs from $26.75 and $2433 per 100 patient days preintervention to $7.14 and $239.80 per 100 patient days postintervention, with p < 0.001. The same study emphasized that a comprehensive multidisciplinary approach must be implemented to decrease inappropriate SUP use in the ICU [9].

This study aimed to appraise the use of SUP in emergency (EICU) and general (GICU) ICUs, assess the costs associated with inappropriate use, and specifically highlight the impact of clinical pharmacists on improving adherence to the SUP guidelines.

## Materials and methods

### Study design and patients

The study is a prospective, non-randomized controlled study. It was conducted for 6 months at the EICU and GICU of a training and research hospital in Türkiye between 1 June 2023 and 1 December 2023.

The study involved GICU patients as the standard care services observation group (OG). The use of SUP in the GICU was only monitored and noted. In the EICU, patients were identified as part of the recruiting guideline group (GG), and in addition to receiving usual care, they were managed for SUP according to the ASHP SUP guidelines [1]. The study duration was 6 months concurrently in the EICU and the GICU. Before inclusion, written informed consent was obtained from each patient or their parent(s)/legal guardian(s).

### Inclusion and exclusion criteria

The inclusion criteria were patients aged ≥18 years and hospitalized for ≥24 h in the EICU/GICU. Patients were excluded from the study if they had a history of stomach cancer, were admitted to the ICU due to GI bleeding, underwent subtotal or total gastrectomy, were on dual antiplatelet therapy, or presented with melena at admission.

### Data collection

Sociodemographic data, medical history (including diseases and drug use), laboratory values (such as platelet count, INR, creatinine, urea, activated partial thromboplastin time, procalcitonin, and C-reactive protein), daily treatment information, culture results, MV status, and scores for the Acute Physiologic Assessment and Chronic Health Evaluation (APACHE2), Sequential Organ Failure Assessment (SOFA), and Glasgow Coma Scale (GCS) were collected from patient follow-up forms and the hospital information management system, with strict adherence to personal confidentiality protocols.

### Assessment of the stress ulcer prophylaxis use

**FIGURE 1:**
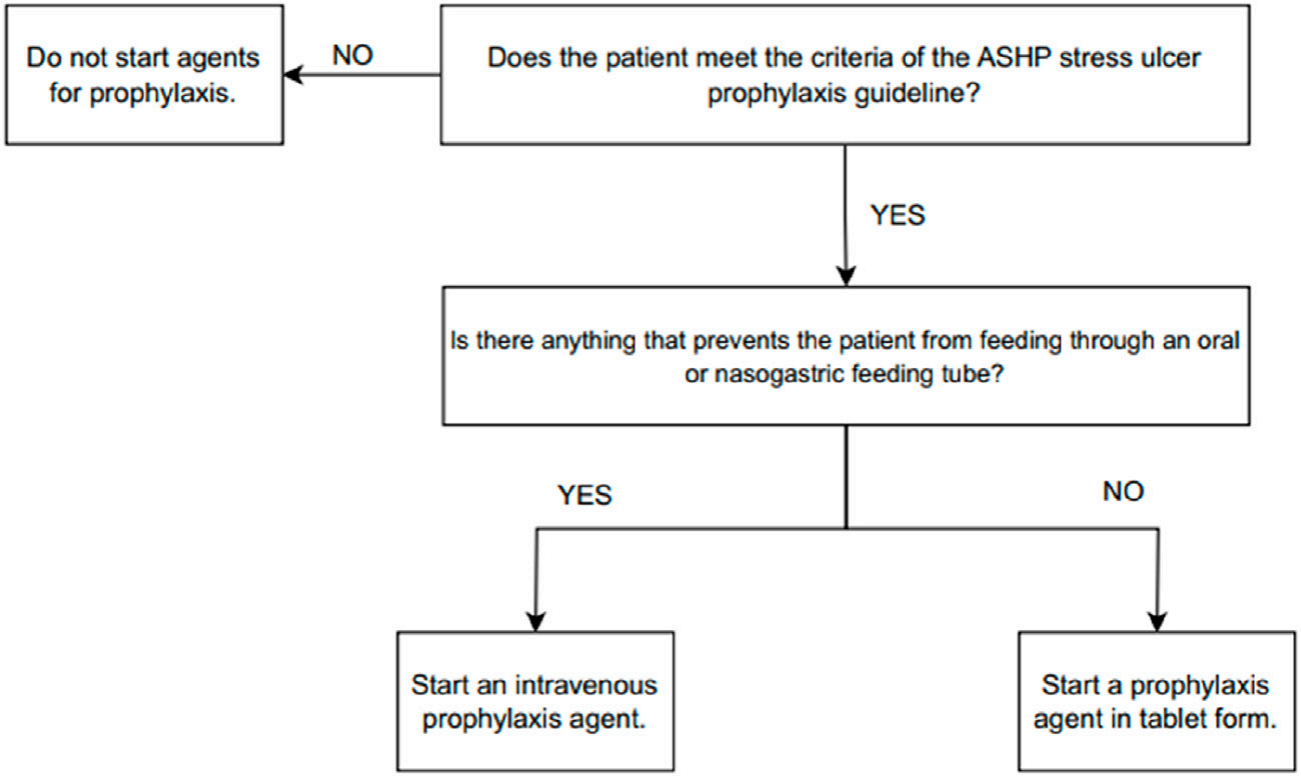
Stress ulcer prophylaxis indication and dosage form suitability.

During weekdays, the researchers monitored and recorded SUP administrations for patients in both the EICU and GICU. The appropriateness of all the patients’ SUP administrations was assessed based on ASHP SUP criteria, considering both the indication and appropriateness of the route of administration [1]. Figure 1 shows the SUP suitability chart assessing the appropriateness of the administration route and dosage form selection for the SUP agent to be given to patients in GG.

In the GG, clinical pharmacists actively participated in patient evaluations, collaborating with physicians to assess the appropriateness of SUP indications and dosage forms based on ASHP criteria. Clinical pharmacists provided real-time recommendations during multidisciplinary rounds, ensuring that only patients meeting guideline criteria received SUP. Additionally, pharmacists educated ICU staff on appropriate SUP use, reinforcing adherence through daily prescription reviews and intervention strategies.

Finally, the specialized physician and clinical pharmacist jointly assessed the appropriateness of the SUP indication for patients and the method of administration. In the final step, the specialist physician evaluated the proper dose and class of the SUP agent for the patients indicated for SUP. Based on the ASHP SUP guidelines, patients who did not meet this indication for SUP did not receive any medication. The patients were observed only in the OG, and the appropriateness of the SUP administrations was documented. Intravenous (IV) SUP agent preparations have been deemed appropriate for patients who cannot take medications orally, who do not have access to enteral nutrition, or who do not have oral intake in cases of gastric hypersecretion associated with neoplastic conditions [13].

According to the ASHP SUP guideline, appropriate SUP use was determined based on the presence of either one major risk factor or two minor risk factors [1]. Patients who met the criteria for either of these groups were considered for appropriate PPI use for SUP.

Major Risk Factors for SUP:

• Coagulopathy: Platelet count <50.000/m^3^, an INR superior to 1.5, or a aPTT superior to 2 times the control value.
• Respiratory failure: The need for mechanical ventilation for at least 48 h.
• Head trauma with GCS ≤10 or inability to follow simple commands.
• Burns involving >35% of total body surface area.
• Partial hepatectomy.
• Liver or kidney transplant.
• Multiple trauma with Injury Severity Score ≥16.
• Spinal cord injury.
• Liver failure.
• History of gastric ulcer or bleeding within the year prior to admission.

Minor Risk Factors for SUP:

• Sepsis.
• ICU stay >1 week.
• Overt or occult bleeding ≥6 days.
• Corticosteroid therapy (daily >250 mg hydrocortisone or equivalent).

### Sample size

Based on statistical calculations using G*Power 3.1.9.7 (Universität Düsseldorf, Germany), with an alpha value of 0.05 and a power of 95%, it was determined that each group should include at least 70 patients. This calculation was based on data demonstrating that clinical pharmacist interventions reduce inappropriate SUP use from 83% to 58% [10]. To account for potential dropouts, the study aimed to enroll a total of 140 participants (70 in each group) to ensure sufficient statistical power. The required sample size was determined using the sample size formula for comparing two independent proportions.

### Definitions

The authors defined significant GI bleeding as bleeding requiring gastroscopy or blood transfusion upon clinician judgment. C. *difficile* infection was defined as the presence of relevant symptoms with positive fecal toxin and/or polymerase chain reaction results in ICU patients after the initiation of SUP in the ICU.

### Outcomes measurement

Adherence rates to ASHP guidelines and costs of nonadherence were primary outcome measurements.

### Data analysis

The descriptive statistics, including the means, medians, standard deviations, interquartile ranges (IQRs), counts, and percentages, were used to assess the central tendency and variability of the continuous variables. For categorical variables, frequencies and percentages are given. The Kolmogorov‒Smirnov test was used to determine whether continuous variables followed a normal distribution. The result was nonparametric. The Mann‒Whitney U test was employed to compare non-normally distributed continuous variables between the two groups, including age, total length of stay, MV duration, SOFA score, APACHE2 score, and GCS score. Categorical data were compared via chi-square tests. A 95% CI with a p value less than 0.05 was considered statistically significant. Analysis of the dataset was performed on an overall basis with the help of IBM SPSS Statistics for Windows, Version 29.0 (Armonk, New York: IBM Corp.).

### Cost savings analysis

This study compared the costs of SUP agents prescribed for inappropriate indications and dosage forms between OG and GG patients. Finally, the SUP cost per patient was determined by multiplying the number of appropriate and inappropriate days of use in both GG and OG by the cost of the dosage form of PPI. The differences in the SUP costs between the two groups are called cost savings.

The costs for the SUP agents were estimated using current drug prices available from the hospital where this study was conducted. Thus, 100 pantoprazole tablets and ten pantoprazole IV ampules were accepted for $1.32 and $2.96, respectively. Only the costs related to PPIs have been calculated. The calculation excluded nursing services and medical supplies.

## Results

**FIGURE 2:**
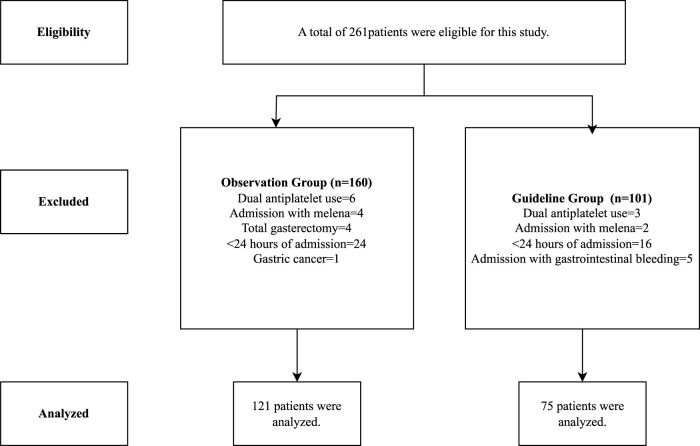
Study’s flowchart.

A total of 261 patients were eligible for the study. Following exclusions, 121 patients remained in the OG and 75 in the GG for analysis, resulting in a total of 196 patients included in the study (Figure 2). Key sociodemographic and clinical characteristics, including age, sex, and comorbidities, were comparable between groups, with differences noted only in median MV duration (10 [5–23] days in OG vs. 0 [0–8] days in GG (p < 0.001) (Table 1).

**TABLE 1:** Sociodemographic information of patients.

Variable	Observation group (n = 121)	Guideline group (n = 75)	Total (n = 196)	p value
Age, median (IQR)	70 (54–81)	67 (50–78)	69 (53–79.75)	0.358
Sex, n (%) Male Female	70 (57.9) 51 (42.1)	37 (49.3) 38 (50.7)	107 (54.6) 89 (45.4)	0.264
Type for hospitalization, n (%) Surgical Medical	60 (49.6) 61 (50.4)	43 (57.3) 32 (42.7)	103 (52.6) 93 (47.4)	0.291
Reason for hospitalization, n (%) Intracranial hemorrhage Chronic obstructive pulmonary disease exacerbation Pneumonia Femur fracture Sepsis Ileus Other	13 (10.7) 7 (5.7) 9 (7.4) 19 (15.7) 13 (10.7) 4 (3.3) 56 (46.2)	10 (13.3) 3 (4) 9 (12) 5 (6.6) 2 (2.6) 0 (0) 46 (61.3)	23 (11.7) 10 (5.1) 18 (9.1) 24 (12.2) 15 (7.6) 4 (2) 102 (52)	-
Comorbidities, n (%) Hypertension Diabetes mellitus Chronic obstructive pulmonary disease Chronic kidney disease Cerebrovascular accident Asthma Coronary artery disease Heart failure Other	67 (29.2) 41 (17.9) 18 (7.8) 12 (5.2) 12 (5.2) 7 (3.0) 22 (9.6) 11 (4.8) 39 (17.0)	39 (28.0) 23 (16.5) 11 (7.9) 6 (4.3) 3 (2.1) 2 (1.4) 10 (7.1) 10 (7.1) 35 (25.1)	106 (20.9) 64 (12.6) 29 (5.7) 18 (3.5) 15 (2.9) 9 (1.7) 32 (6.3) 21 (4.1) 74 (14.5)	-
Discharged status, n (%) Discharged Death	104 (86) 17 (14)	65 (86.7) 10 (13.3)	169 (86.2) 27 (13.8)	0.898
BMI, median (IQR), (kg/m^2^)	25.5 (22.4–29.4)	26.2 (22.8–30.8)	25.8 (22.8–29.7)	0.331
Total length of stay, median (IQR) (day)	4 (1–11.5)	2 (1–11)	3 (1–11)	0.358
SOFA score, median (IQR)	2 (1–5)	2 (1–4)	2 (1–5)	0.691
APACHE2 score, median (IQR)	13 (9–18)	13 (8–18)	13 (9–18)	0.605
Hemodialysis status, n (%) Yes No	1 (0.8) 120 (99.2)	0 (0) 75 (100)	1 (0.5) 195 (99.5)	0.430
GCS score, median (IQR)	15 (10–15)	15 (12–15)	15 (10.25–15)	0.385
eGFR, median (IQR)	91 (66–113.5)	89 (67–100)	89 (66–106)	0.687
MV status, n (%) Yes No	41 (33.9) 80 (66.1)	25 (33.3) 50 (66.7)	66 (33.6) 130 (66.4)	0.937
MV duration, median (IQR) (day)	10 (5–23)	0 (0–8)	2 (0–12)	<0.001
Nutrition type, n (%) Oral Nasogastric feeding tube	64 (52.8) 57 (47.2)	47 (62.6) 28 (37.4)	111 (56.6) 84 (42.8)	0.322

APACHE, Acute Physıologıc Assessment And Chronıc Health Evaluatıon; BMI, Body mass index; GCS, Glasgow coma scale; eGFR, Estimated glomerular filtration rate; IQR, Interquartile range; LFTs, Liver function tests; MV, Mechanical ventillation; SOFA, Sequential Organ Failure Assessment.

SUP was administered to all patients in the OG and 42.6% of patients in the GG (p < 0.001). Adherence to ASHP guideline indications was significantly higher in the GG (100%) than in the OG (54.5%, p < 0.001). Dosage form adherence was significantly lower in the OG (0%) than in the GG (100%), with all patients in the OG receiving IV PPIs. C. *difficile* infection and GI bleeding were not encountered in the patients included (Table 2).

**TABLE 2:** Stress ulcer prophylaxisuse and adherence with the ASHP stress ulcer prophylaxis guideline.

Variable	Observation group (n = 121)	Guideline group (n = 75)	Total (n = 196)	p value
Stress ulcer prophylaxis use, n (%) Yes No	121 (100) 0 (0)	32 (42.6) 43 (57.3)	153 (78) 43 (22)	<0.001
Indication^a^, n (%) Appropriate Inappropriate	66 (54.5) 55 (45.5)	32 (100) 0 (0)	98 (50) 55 (28)	<0.001
Dosage form/route of administration^a^, n (%) Appropriate Inappropriate	0 (0) 121 (100)	32 (100) 0 (0)	32 (20.9) 121 (79.1)	<0.001
Number of indications^b^, n (%) 1 2 3 4	24 (36.3) 33 (50) 8 (12.2) 1 (1.5)	14 (43.7) 17 (53.12) 1 (3.1) 0 (0)	37 (38.1) 50 (51.5) 9 (9.4) 1 (1)	0.068
Stress ulcer prophylaxis criteria^b^ Major criteria Coagulopathy ≥48 h MV Head injury with GCS ≤ 10 Liver or kidney transplant Polytrauma with Injury Severity Score ≥ 16 Spinal cord injury Liver failure Partial hepatectomy Burns History of gastric ulcer or bleeding Minor criteria Sepsis >1 week intensive care unit stay Occult or overt bleeding for ≥ 6 days, Corticosteroid treatment	6 (5.1) 48 (41) 31 (26.4) 0 (0) 5 (4.2) 1 (0.8) 2 (1.7) 0 (0) 0 (0) 0 (0) 24 (20.5) 12 (10.3) 0 (0) 12 (10.3)	3 (5.8) 22 (43.1) 15 (29.4) 1 (1.9) 5 (9.8) 0 (0) 0 (0) 0 (0) 0 (0) 0 (0) 5 (9.8) 4 (7.8) 0 (0) 1 (1.9)	9 (5.3) 70 (41.6) 46 (27.3) 1 (0.5) 10 (5.9) 1 (0.5) 2 (1.1) 0 (0) 0 (0) 0 (0) 29 (17.2) 16 (9.4) 0 (0) 13 (7.6)	-
Gastrointestinal bleeding, n (%) Yes	0 (0)	0 (0)	0 (0)	>0.05
*C. difficile* infection, n (%) Yes	0 (0)	0 (0)	0 (0)	>0.05

GCS, Glasgow coma scale; MV, Mechanical ventillation; SUP, Stress ulcer prophylaxis.

^a^ PPI was not used in 43 patients because there was no indication.

^b^ Patients had more than one indication.

Inappropriately indicated SUP was observed only with pantoprazole in patients. The costs of PPI use with inappropriate indications and inappropriate dosage forms in OG and GG were $60 vs. $0, and $321 vs. $0, respectively (p < 0.001). Accordingly, the total cost savings were calculated to be $327 (Table 3).

**TABLE 3:** Distribution of costs of stress ulcer prophylaxis between groups.

Variable	Observation group (n = 121)	Guideline group (n = 75)	*p* value
Total/per patient (dollars) Appropriate indication Inappropriate indication	262/2.1 60/0.5	6/0.08 0/0	0.001
Total/per patient (dollars) Appropriate dosage form Inappropriate dosage form	0/0 321/2.65	6.2/0.08 0/0	0.001

In OG patients, those with an appropriate SUP indication had significantly higher SOFA and APACHE2 scores, indicating greater illness severity compared to those with an inappropriate indication, as presented in Table 4 (p < 0.001). They also had a longer hospital stay, suggesting a more complex clinical course. In terms of discharge status, mortality was observed only in the appropriate group, while all patients in the inappropriate group were discharged (p < 0.001). For MV, nearly all ventilated patients were in the appropriate indication group, while the majority of non-ventilated patients were in the inappropriate group (p < 0.001). When evaluating reasons for hospitalization, medical conditions were more frequent in the appropriate group, whereas surgical cases were predominant in the inappropriate group (p < 0.001).

**TABLE 4:** Statistical analysis of SUP appropriateness with associated data in observation group.

Variable	Indication		p value
	Appropriate (n = 66)	Inappropriate (n = 55)	
SOFA score, median (IQR)	4 (2–6.5)	1 (0–2)	<0.001
APACHE2 score, median (IQR)	17 (13–22)	10 (7–13)	<0.001
Total length of stay, median (IQR) (day)	10.5 (5–24.5)	1 (1–1)	<0.001
GCS score, median (IQR)	12.5 (4.5–15)	15 (15–15)	<0.001
Discharged status, n (%) Discharged Death	49 (74.2) 17 (25.8)	55 (100) 0 (0)	<0.001
Nutrition type, n (%) Oral Nasogastric feeding tube	15 (22.7) 51 (87.3)	49 (89) 6 (11)	<0.001
Mechanical ventillation status, n (%) Yes No	40 (60) 26 (40)	1 (0.01) 54 (99.99)	<0.001
Reason for hospitalization, n (%) Surgical Medical	16 (24.2) 50 (75.8)	44 (80) 11 (20)	<0.001

APACHE, Acute Physıologıc Assessment And Chronıc Health Evaluatıon; GCS, Glasgow coma scale; IQR, Interquartile range; SOFA, Sequential Organ Failure Assessment.

Factors influencing appropriate SUP prescriptions in the OG included ICU admission for medical reasons (OR: 0.08, 95% CI: 0.034–0.191), ICU stay >4 days (OR: 0.008, 95% CI: 0.002–0.032), and severity of illness as indicated by SOFA scores >2 (OR: 0.070, 95% CI: 0.028–0.177) and APACHE2 scores >13 (OR: 0.105, 95% CI: 0.045–0.242) (all p < 0.001). Patients with a GCS score less than 15 had a significantly high OR of receiving appropriate SUP: OR 24.7, CI 95%, p < 0.001. MV has a significantly high OR for proper SUP utilization: 83.07, CI ranging from 10.81–638, p < 0.001. Moreover, nasogastric feeding tube delivery of nutrition and the development of mortality emerged as significant variables for receiving SUP, with ORs of 0.036 and 0.471, respectively, with 95% CIs of 0.13–0.1 and 0.384–0.578, respectively (p < 0.001) (Table 5).

**TABLE 5:** Analysis of relative risk factors for stress ulcer prophylaxis in appropriate indication in observation group.

Risk factors	OR (95% CI)	*p*
ICU admission for medical reasons	0.08 (0.034–0.191)	<0.001
>4 days ICU stay	0.008 (0.002–0.032)	<0.001
Death	0.471 (0.384–0.578)	<0.001
>2 SOFA score	0.070 (0.028–0.177)	<0.001
>13 APACHE2 score	0.105 (0.045–0.242)	<0.001
<15 GCS score	24.7 (8.5–71.5)	<0.001
Nutrition via nasogastric feeding tube	0.036 (0.13–0.1)	<0.001
Mechanical ventillation	83.07 (10.81–638)	<0.001

APACHE, Acute Physıologıc Assessment And Chronıc Health Evaluatıon; CI, Confidence interval; GCS, Glasgow coma scale; ICU, Intensive care unit; OR, Odds ratio; SOFA, Sequential Organ Failure Assessment.

## Discussion

In this study, we assessed the use of SUP in the EICU and GICU, determined the costs of inappropriate use, and highlighted the impact of clinical pharmacists on improving adherence to the SUP guidelines. SUP is frequently prescribed in the ICU to decrease the incidence of GI bleeding. Different studies have revealed that the rate of SUP utilization in ICUs is between 81.2% and 92.9% [14–17].

The published literature has varied the rates of adherence to SUP prescriptions in ICUs, thereby showing changes in procedures and guidelines, especially those designed by the ASHP. Various studies have reported rates of inappropriate SUP prescriptions that do not fall within the criteria set in the guidelines of 58–68.1% [6, 14–16, 18, 19]. In contrast to our study, which reported 45.5% inappropriate SUP prescriptions, other studies reported a lower rate of inappropriate SUP prescriptions, ranging from 14% to 38.5% [6, 10, 17, 20]. A recent study conducted in Türkiye using a pre- and post-education design in the ICU reported inappropriate SUP usage rates of 61.7% and 52.2%, respectively [21]. It was common practice in the center where this study was conducted to prescribe PPIs for SUP to every patient admitted to the ICU at a rate higher than that documented in the literature. Despite dispensing SUP without the influence of any specific guidelines or protocols, the adherence rate of the OG to the ASHP guidelines was within the literature range [6, 14–16, 18, 19]. Several factors might have contributed to the different rates of inappropriate SUP use in this study compared with those in other studies. These factors include the type of hospital, disparities between admissions of medical and surgical patients in the ICU, and assessment of appropriateness by different guidelines and protocols [10, 20, 22]. Thus, based on these studies, one assumes that there is a widespread problem of excessive SUP prescription in ICUs.

The use of IV dosage forms for SUP in ICU patients is recommended not only based on the appropriateness of indications but also due to clinical circumstances requiring intravenous administration [14, 23, 24]. Recent evidence has established the efficacy of IV PPI preparations in patients with gastric hypersecretion and Zollinger–Ellison syndrome associated with neoplastic conditions, those who are unable to take oral medications, those with severe nonvariceal upper GI bleeding, those with GI bleeding with a high risk of recurrent or continuous bleeding, and ICU patients who do not have access to enteral nutrition or oral intake. IV PPI is particularly indicated in such high-risk patients [13]. However, some studies have shown that IV PPI is abused, especially when there is no significant indication of upper GI bleeding [13, 24, 25]. Inappropriate IV dosage rates for SUP have been reported 19.8%–33.3% [13, 23, 26]. Hoover et al. concluded that IV pantoprazole rather than oral esomeprazole was deemed inefficient, showing matters related to both cost-effectiveness and procedural application [23]. Although all of the patients within the OG were qualified to prescribe drugs by oral or nasogastric feeding tube route, all patients were prescribed an IV PPI, of which none were qualified.

In the GG, following the guidelines and considering dosage forms, we concluded that prescription PPIs in oral or nasogastric tube form, specifically as tablets, for all patients are enough. The data obtained from the studied hospital’s ICUs regarding inappropriate and frequent prescribing of IV PPIs points to a habitual practice that must be changed. Emphasizing the cost difference between IV and oral PPIs could help address this issue. Many studies emphasize the collaborative role of clinical pharmacists in better adherence to SUP guidelines through active management. According to the literature, clinical pharmacists are essential and efficient in prescribing SUP. Key issues that have been raised include pharmacists’ involvement in visits to the patient, conducting training programs, and making decisions with physicians to optimize SUP practice. The active involvement of clinical pharmacists played a key role in improving adherence to SUP guidelines. Their presence in multidisciplinary rounds facilitated real-time decision-making, reducing inappropriate SUP prescriptions. Previous studies have highlighted that pharmacist-led interventions, including direct physician education and medication reviews, can significantly enhance guideline adherence. Our findings further support this, demonstrating that pharmacist-guided prescribing led to better compliance with ASHP criteria, reduced unnecessary IV PPI use, and contributed to cost savings. Implementing similar pharmacist-led strategies in other ICUs could help optimize SUP practices and minimize medication-related risks. Hammond et al. illustrated the powerful positive influence of pharmacist-physician collaboration in the ICU to improve compliance with SUP prescribing guidelines through a structured educational intervention. The authors noted that this cost-effective measure could easily be extrapolated to facilities where pharmacists participate in rounds with physicians [27]. Similarly, Mahmoudi et al. evaluated the appropriateness of SUP by applying ASHP criteria and studied the economic effects of clinical pharmacist interventions. Their study revealed a significant cost savings of more than $18,000 per month from clinical pharmacists’ interventions [17]. Rafinazari et al. performed a similar study in which they concluded that educating physicians about the proper implementation of standard protocols and developing collaborations with clinical pharmacists could result in improved prescribing practices for SUP [6]. Consequently, it results in a relative reduction in hospital expenditures and an absolute reduction in hospital costs and adverse drug reactions.

Various strategies have been proposed to address the inappropriate use of SUPs. Some have resident training as their component, while some pharmacist-based strategies have also been proposed with encouraging results [10, 11, 28]. Buckley et al. developed a pharmacist-based strategy that reduced inappropriate prescribing of SUPs [10]. With the exceptions of the studies of Buckley et al. and Masood et al., all the studies relied on pharmacists educating physicians regarding appropriate SUP use rather than empowering them to prescribe it [9, 10]. On the other hand, Buckley et al. created a program that pharmacists ultimately drive without institutional staff input at academic facilities [10]. Masood et al. developed a two-stage system that involves a review during ICU team visits, including the pharmacy team, and another review of treatments after the visits [9]. In this study, patients in GG benefit from collaboration with physicians and clinical pharmacists, who jointly operate according to the ASHP guidelines and dosage form appropriateness. With respect to appropriate medication intervention, the rate of SUP prescriptions and forms used with inappropriate dosing in the ICU was significantly lower than that in the OG.

The study’s methodology explicitly addresses the cost status of drugs to accurately reflect the discrepancy between IV and oral pantoprazole, considering the high dollar exchange rate against the Turkish lira. As a result, the substantial costs associated with inappropriate use of the IV dosage form in patients have also been minimized. Because the cost-saving computations include only patients in the GG and IV-oral pantoprazole groups, the total cost reduction may appear minimal. Although the cost savings varied in most of the studies where the clinical pharmacist was involved in increasing SUP appropriateness via different strategies, this study confirmed that the inclusion of the clinical pharmacist on the team contributed to cost reduction.

Some studies aimed at reducing inappropriate SUP use may also inadvertently decrease appropriate use, potentially increasing the risk of stress ulcers and related complications. However, adherence to guidelines helps prevent unnecessary adverse effects of SUP medications. In Anstey et al.'s study, which sought to apply the SUP protocol, the frequency of C. *difficile* linked to PPIs dropped from one patient out of ten in the pre- and post-implementation groups [22]. Masood et al. noted that, due to their study’s limitations, they could not follow patients for GI bleeding or C. *difficile* infections [9]. In our study, no GI bleeding or C. *difficile* infection was detected in the guideline group, indicating that adherence to ASHP guidelines contributed to reducing unnecessary prescriptions and costs. However, the absence of a dedicated control group limits our ability to conclusively determine the effect of guideline adherence on adverse events. Further randomized controlled studies are needed to explore the causal relationships among PPI use, GI bleeding, and C. *difficile* infections [29]. While our study demonstrates that adherence to guideline-based SUP strategies can reduce inappropriate prescribing and costs, long-term clinical implications require further investigation. Although no cases of GI bleeding or C. *difficile* infections were observed in our study, longer follow-up periods are necessary to determine whether reducing inappropriate SUP use impacts these clinical outcomes. Additionally, ICU length of stay is a critical factor in SUP decision-making. Our findings indicate that patients receiving appropriate SUP had longer ICU stays, likely reflecting their greater severity of illness rather than an effect of SUP itself. Future studies should explore whether optimizing SUP prescribing influences ICU length of stay, hospital-acquired infections, and overall patient prognosis.

Moreover, the literature consists of studies that determine predictors for inappropriate, excessive usage of SUP in the ICU. These predictive factors are age, sex, length of hospital stay, reason for admission to the medical-surgical ICU, and educational status regarding SUP [15, 19, 30, 31]. The length of hospital stay and the number of comorbidities were identified as risk factors by Issa et al. [19]. Alsultan et al. did not find a link between SUP use and hospital stay duration; however, Mayet et al. reported that appropriate acid suppression treatment rates increased with longer lengths of stay [13, 32]. Moreover, some studies have shown that increasing patient age and sex predict inappropriate PPI use [24, 31, 33, 34]. However, a more recent study has shown that the appropriateness of PPI treatment in patients is not influenced by sex [13, 32]. Factors indicating a poor prognosis, for example, a high APACHE2 score, a high SOFA score, a low GCS score, the presence of MV, nasogastric tube feeding, an extended length of stay, and hospitalizations ending in death, are associated with a significant likelihood of the prescription of SUP according to the guidelines used in this research. Patients with an extended ICU stay are sicker in terms of their underlying medical condition, are more prone to developing a greater number of ICU-related complications and often have a poor prognosis. As a result, the longer the duration of stay is, the more familiar the major and minor criteria for SUP are, making it very common and appropriate in this subset of patients.

In this study, the duration of MV was significantly longer in the OG compared to the GG. When evaluating the admission types (surgical vs. medical) of patients in the EICU and GICU (Table 1), it was observed that medical admissions were more prolonged in GICU patients within the OG. Additionally, EICU patients had shorter hospital stays, fewer comorbidities, and different reasons for admission compared to GICU patients. Despite MV duration exceeding 48 h being a key criterion for appropriate SUP use, the rate of adherence to SUP guidelines was notably low in the OG. One would expect a higher adherence rate in a group where such a fundamental criterion differed significantly. However, in contrast, appropriate SUP prescribing was observed at a higher rate in the GG, where clinical pharmacists played a role in ensuring adherence to the ASHP guidelines.

Moreover, clinically significant bleeding is unlikely to occur in postoperative patients, and the use of SUP in such cases is controversial [34]. In the present study, inappropriate prescription of SUP was more likely to occur during surgical stays. As has been the case in other studies, this study did not find that age and gender significantly affected the appropriateness of SUP. In this regard, some studies suggesting that gender significantly affects SUP use do not have any rational justification [30, 32, 33]. Moreover, inappropriate SUP prescriptions may be influenced by education. Recent studies have reported more guideline-adherent SUP prescriptions in academic institutions than in nonacademic hospitals [35–37].

This study has several strengths, including its clinical relevance in addressing inappropriate SUP use in ICUs, a structured prospective design that allows meaningful comparisons, and the interdisciplinary involvement of clinical pharmacists, which significantly improved guideline adherence. The robust statistical analysis and detailed cost evaluation further strengthen the study by demonstrating clear financial benefits. Additionally, this study is one of the few conducted in the country with this specific design and focus on SUP adherence in the ICU setting. By integrating clinical pharmacists into ICU practice, our findings provide a practical, cost-effective strategy that can be adapted in other healthcare centers to enhance adherence to SUP guidelines and reduce unnecessary prescriptions.

However, certain limitations must be acknowledged. First, the non-randomized design may introduce selection bias, potentially affecting the outcomes. Second, as a single-center study, the generalizability of the results to other hospitals and ICU settings may be limited. Additionally, the six-month study period may not fully capture long-term prescribing trends, cost variations, or the sustained impact of clinical pharmacist interventions. While this study successfully demonstrates cost savings and improved guideline adherence, it does not assess long-term patient outcomes, such as the incidence of stress ulcers or adverse drug reactions, which would provide further clinical insights. Future randomized controlled trials with longer follow-up periods are needed to evaluate the sustained effects of guideline-based SUP interventions on both clinical outcomes and healthcare costs. Although the sample size was statistically powered using G*Power analysis, a larger patient cohort over an extended period could have further strengthened the findings. Extending the study duration would have allowed for a broader patient inclusion, but logistical challenges, such as ICU workload and coordination among the clinical team, limited the feasibility of a longer enrollment period.

The cost analysis in this study was deliberately focused on drug costs to highlight the direct financial impact of inappropriate SUP prescribing. While additional ICU-related expenses—such as prolonged hospital stays, nursing care, unnecessary IV administration supplies (syringes, gloves, IV sets), and laboratory tests—could further increase total cost savings, calculating these elements in detail may have made the analysis overly complex and less practical. However, incorporating such indirect costs in future studies could provide a more comprehensive economic evaluation.

Physician prescribing habits significantly influence SUP use, and institutional prescribing patterns may have affected our findings. While the study was conducted in an ICU with historically high SUP use, the effect of pharmacist-led interventions may vary in settings where inappropriate prescribing rates are lower. However, SUP overuse and non-adherence to guidelines have been widely reported in ICUs both nationally and globally, suggesting that our findings remain relevant beyond the study setting. Future multicenter studies are needed to validate these results in different hospital environments with varying prescribing practices.

## Conclusion

This study, which is focused on local practices, reflects the international problem of excessive and inappropriate SUP use. Naturally, overcoming this challenge without cooperation between the clinical pharmacist and the physician is not feasible. This collaboration reduced PPI use and associated costs while promoting safe, cost-effective SUP practices through increased guideline adherence.

## Data Availability

The raw data supporting the conclusions of this article will be made available by the authors, without undue reservation.
